# Deuterium Distribution in Fe/V Multi-Layered Films

**DOI:** 10.3390/molecules27227848

**Published:** 2022-11-14

**Authors:** Ryota Gemma, Talaat Al-Kassab, Astrid Pundt

**Affiliations:** 1 Department of Applied Chemistry, Tokai University, Hiratsuka 259-1292, Japan; 2 Micro/Nano Technology Center, Tokai University, Hiratsuka 259-1292, Japan; 3 Institute of Materials Physics, University of Göttingen, 37077 Göttingen, Germany; 4 Institute for Applied Materials–Materials Science and Engineering (IAM-WK), Karlsruhe Institute of Technology, 76131 Karlsruhe, Germany

**Keywords:** atom probe, hydrogen, deuterium, interface

## Abstract

The recent progress of Atom Probe Tomography (APT) has opened up atomic-scale elemental analysis including hydrogen species. For APT measurements, the use of deuterium is highly recommended, due to its low mobility compared to the fast and quantum mechanically tunneling isotope hydrogen. In addition, deuterium can be distinguished from hydrogen originating from the APT analysis chamber. To date, however, APT studies on materials with high D concentrations are scarce. In this study, the D concentration profile in a Fe/V multi-layered film sample was investigated, and spanned a wide concentration range. The mean hydrogen isotope concentration was alternatively quantified by electromotive force (EMF) measurements on a similar Fe/V film, thus verifying the APT results. The reduction found in the D concentration at the Fe/V interface results from local alloying at the Fe/V interfaces which accompanies a change in the available volume in the V lattice. Even at the same Fe concentration, the shape of the observed D depth profile was asymmetric at high D_2_ pressures. This indicates a stress impact caused by the deposition sequence.

## 1. Introduction

Atom Probe Tomography (APT) has been well recognized as a strong materials analysis tool to investigate three-dimensional elemental distribution at a spatial resolution at the sub-nm scale [1,2,3,4,5,6,7,8]. The beauty of APT lies in the ability of detecting all elements with equal sensitivity at once, as it is based on the time-of-flight principle. Fine-scale 3D mapping of hydrogen in real space [9,10] is highly motivated by, e.g., studies of hydrogen embrittlement in metallic materials [11,12,13] and the impact of hydrogen in semiconductors [14,15,16]. APT is also used for developing hydrogen storage materials and their potential applications in catalytic reactions such as CO_2_ methanation [17]. Detection of hydrogen and its mapping, however, has been a challenging task for a few decades since the invention of APT. The difficulties stem from (1) the high mobility of hydrogen, (2) hydrogen loss from the specimen by air exposure, and (3) the necessity to distinguish loaded ions from residual hydrogen ions present in the APT analysis chamber.

Recent studies and developments of APT analysis have made significant progress to tackle the above-mentioned issues, e.g., by using deuterium (D) instead of hydrogen (H), and by establishing a loading/transfer method, which is free from air exposure of the specimen. Successful adaption of these protocols enabled quantification of D in, e.g., V [18], which easily absorbs hydrogen isotopes (the Gibbs free energy change, Δ*G*, of the hydrogen solid solution reaction at room temperature is negative). However, in a material such as iron or steel that hardly dissolves hydrogen isotopes (Δ*G* at room temperature is largely positive), the driving force for hydrogen desorption is large even when placed in an ultra-high vacuum. D easily escapes during sample transfer under vacuum in this case.

In order to suppress the diffusion and desorption of D, it is therefore necessary to keep the sample at a cryogenic temperature after D charging until the analysis is carried out. In this regard, the first attempt was reported by Takahashi et al. [19] for investigating D trapping around precipitates. Chen et al. [20,21] have applied a cold-chain approach on electrochemically deuterated steel samples. Stephenson et al. [22] have used a similar method and, thus, opened up the APT technique for a quantitative analysis of D distribution in steel samples. Quite recently, Felfer et al. [23] have proposed using an APT chamber made of Ti in order to suppress H-related signals and to ultimately detect H in the specimen.

We have previously reported our first quantitative APT analysis results of different D contents solved in samples. This was achieved using Fe/V multi-layered (ML) films [24,25] and V films [18]. According to the knowledge of the solubility difference factor, *k*, the solubility of D in V is 10^10^ higher than that in Fe at room temperature [26]. Therefore, almost all of the D atoms are expected to be present in the V layer. This was indeed experimentally observed by the APT analysis in agreement with the expected mean D concentration in V layers measured by the electromotive force (EMF). Thereby, it was demonstrated that the mean D concentration by APT could be successfully quantified as long as the D concentration is low, i.e., within the solid solution regime. However, similar results of APT studies with a high D concentration in Fe/V ML have not been reported yet.

In addition, the local D distribution might not be homogeneous in Fe/V films at high D concentrations. In bulk V, available sites for H change from the tetrahedral site (T site) to the octahedral site (O site) by increasing H concentration and also by changing initial lattice strain [27]. H in thin films is typically subject to this strain impact, as they are often adhered to rigid substrates. Pálsson et al. [28] experimentally observed a strain-related change in the site occupancy, from the T_xy_ site to the O_z_ site in the out-of-plane direction, for Fe/V (001) superlattices. This strain-sensitive effect was further supported by DFT calculations of Johansson et al. [29]. Such site occupancies can not be addressed by APT measurements, but local concentrations can be targeted. In this contribution, we present APT analysis results of D distributions in Fe/V ML samples loaded at different D_2_ pressures that relate to high D contents, with paying attention not only to the mean D concentration in the V layers, but also to the D concentration profile at Fe/V interfaces.

## 2. Results and Discussion

### 2.1. Characterization of Fe/V ML Interfaces

#### 2.1.1. Layer Interdiffusion at a High Deposition Temperature

**Figure 1:**
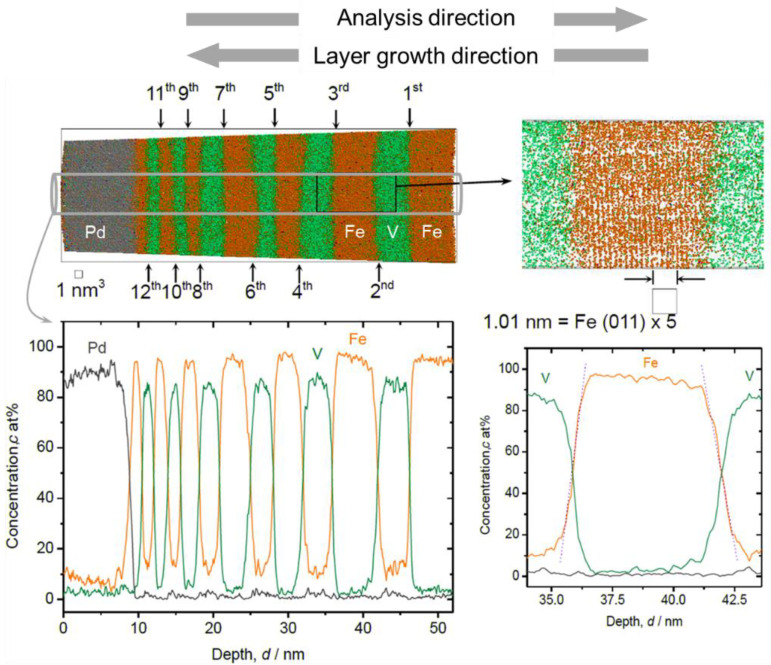
3D reconstruction volume (18 nm × 18 nm × 53 nm) and 1D concentration profile of Fe/V multi-layered film on W deposited at 603 K (grey: Pd, green: V, orange: Fe), with a magnified V/Fe/V interface (right hand side). The number of Fe/V interface (12 in total) is indicated as the order number. Depth concentration profiles were taken from a 5 nm *ϕ* cylinder volume (colored in grey).

The local chemistry at the Fe/V interface is one of our interests in this study, particularly regarding its relationship to the D concentration profile. For epitaxial growth of hetero-structures, deposition at high temperature is often conducted to obtain smooth interfaces [30]. In this study, sputter deposition of Fe/V multi-layer was carried out also at a high temperature, 603 K, as a trial. An APT reconstruction result of a thickness-variated Fe/V multi-layered stack deposited at 603 K is shown in Figure 1, together with the depth concentration profile taken from a 5 nm *ϕ* cylinder volume.

Clearly resolved parallel Fe (011) planes prove a result of successful APT reconstruction and a growth with (110) texture through the Fe/V ML. The Fe content (*c*_Fe_) found in the V layers was higher than 10 at%. This value is higher than that expected from our experimental condition, which is in the range of 2~8 at%. Therefore, other processes, such as interdiffusion due to high temperature deposition or intermixing by sputtering are responsible to increase the Fe content. It should also be noted that the alloying of V with Fe becomes significant towards the surface and also high Fe content is detected in the Pd surface layer.

**Figure 2:**
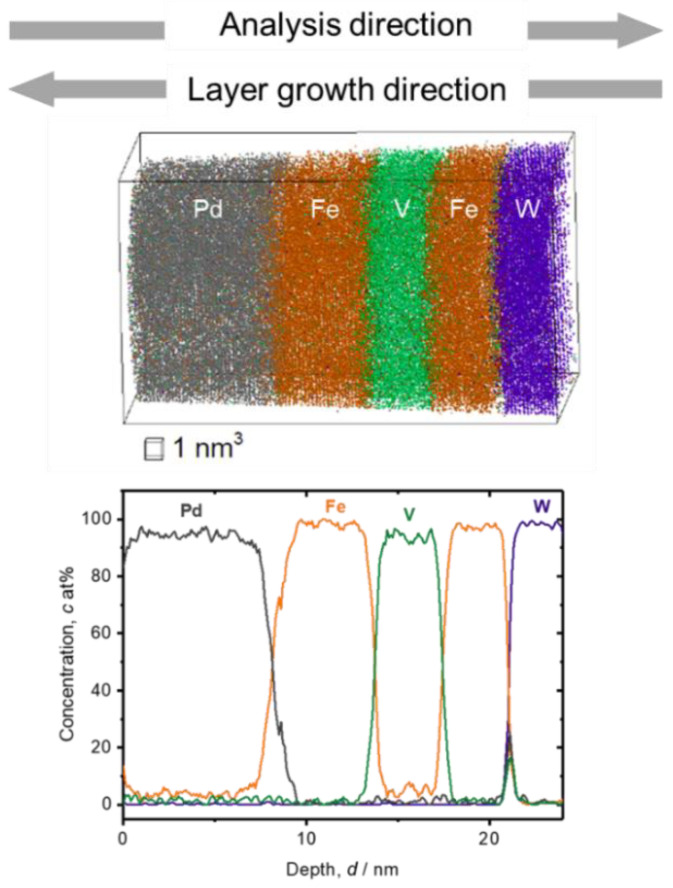
3D reconstruction volume (13 nm × 13 nm × 26 nm) and 1D concentration profile of Fe/V multi-layered film deposited on W deposited at 297 K (grey: Pd, green: V, orange: Fe, blue: W). Depth concentration profile was taken from a 5 nm *ϕ* cylinder volume. The slope for deposition sequence of V on Fe is slightly steeper than that of Fe on V.

Alternatively, when the Fe/V multi-layered film is deposited at room temperature, both the Fe concentration in V and vice versa are rather low, as shown in the APT analysis result of Figure 2. The Fe concentration in V layer is, in this case, 5(2) at% and the V concentration in Fe is nearly zero. The Fe concentration is high (10 at%) at the top surface of Pd, which is found also for the film deposited at 603 K. The slope of the Fe/V interfaces is sharper than in the case of 603 K.

**Figure 3:**
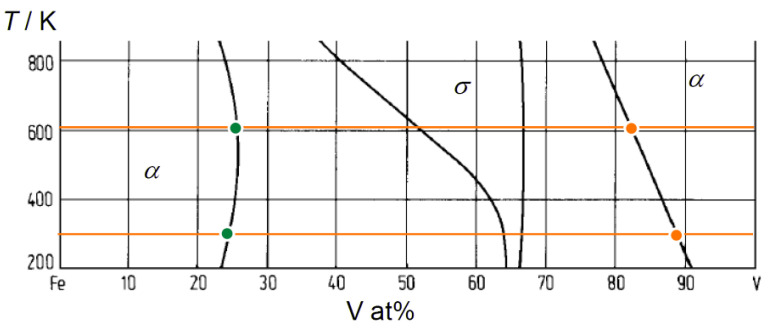
Low temperature part of Fe-V binary phase diagram [31]. 603 K and 297 K are indicated by horizontal orange lines. Fe solubility limit (orange) and V solubility limit (green) at each temperature are marked by individual filled circles.

The Fe and V concentrations in the layers can be discussed with respect to the Fe-V phase diagram. The low temperature part of the Fe-V binary phase diagram [31] is shown in Figure 3. The solubility of Fe in V and V in Fe at 603 K is 17 at% and 26 at%, respectively. At 297 K, they reduce to 12 at% and to 25 at%, respectively. Further excess causes the precipitation of σ phase. The detected Fe and V concentrations were after all below the solubility limits and, therefore, only the α solid solution phase is considered. Consequently, the observed high Fe content in the V layers could be caused by thermal interdiffusion at 603 K, while it is suppressed at 297 K.

However, there are following points left to be clarified and discussed in the next section.

- Origin of asymmetric Fe/V interface profile at an elevated deposition temperature, and
- Increasing alloying degree towards the surface.

#### 2.1.2. Interface Intermixing by Sputtering Process

**Figure 4:**
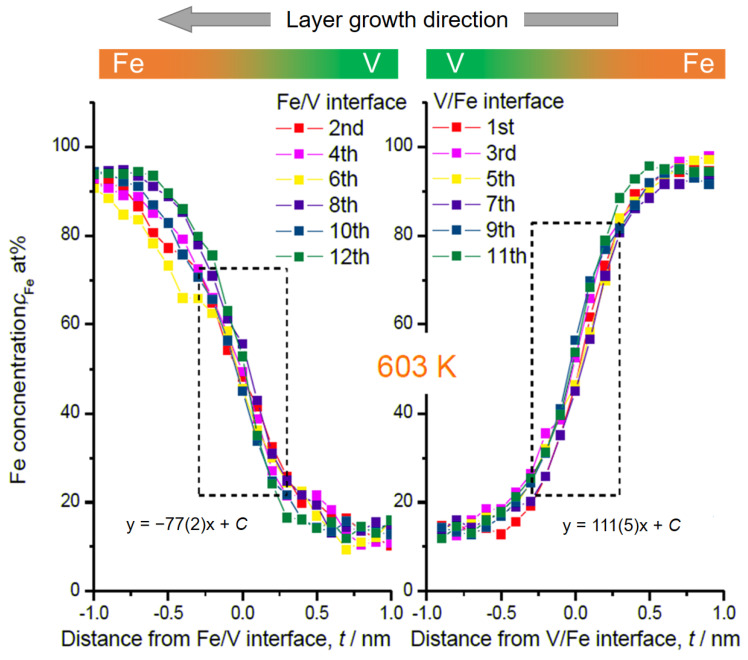
Fe concentration profiles at Fe/V and V/Fe interfaces in Figure 1. The numbers are assigned as for the interfaces indicated in Figure 1. The dotted rectangular region indicates where the interface sharpness was estimated as a slope. The concentration slope at each in the same series is almost identical. But the average slope for deposition sequence of V on Fe (111(5)) is steeper than that of Fe on V (−77(2)).

**Figure 5:**
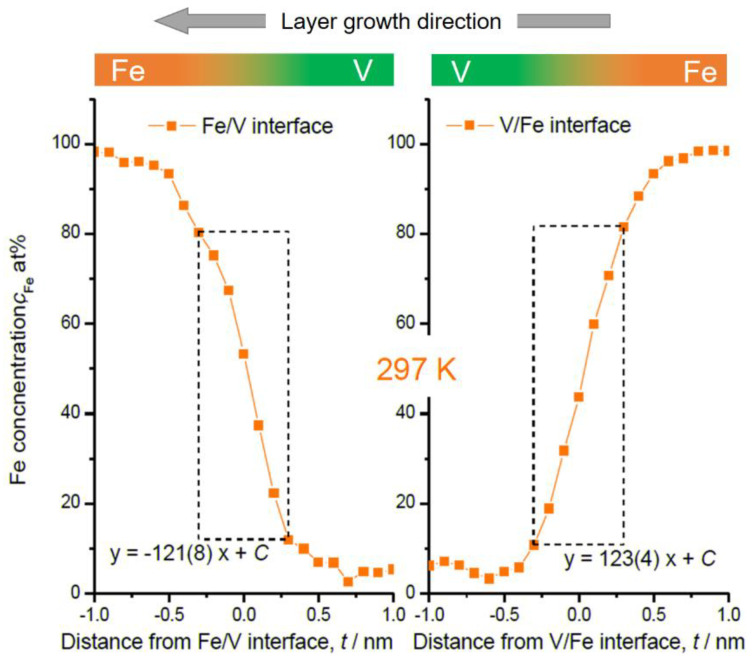
Fe concentration profiles at Fe/V and V/Fe interfaces in Figure 2. Dotted rectangular region indicates where the interface sharpness was estimated as a slope. The concentration slopes estimated within the dotted squares are almost the same.

In Figure 4 and Figure 5, the magnified V/Fe/V region is shown for the films deposited at 603 K and at 297 K, respectively. According to these profiles, depending on the sequence, the intermixing depth can be estimated as wide as 2 nm when deposited at 603 K (Figure 4). This value is almost twice as the mixing regime at 297 K, which is only 1.0~1.1 nm (Figure 5).

In Figure 4, some changes in the concentration slope can be seen between the 2nd and the 6th Fe/V interface. However, there is no systematic change in the slope in the course of deposition even at the high temperature of 603 K. Therefore, interdiffusion processes solely cannot be convincingly attributed for the explanation. We should note that the field evaporation sequence in atom probing can also be affected by local chemistry via modification of the field evaporation strength *E_F_*. The change in *E_F_* sometimes induces a significant deviation from the original position of atoms. In this case, special care must be taken by examining the interface of two different layers [32,33,34]. Indeed, the *E_F_* of Fe^2+^ (30 V/nm) is slightly lower than that of V^2+^ (33 V/nm) [3], which leads to preferential evaporation of Fe while retarding the evaporation of V at a given field. This possibly causes reduced Fe concentration in an evaporation sequence of Fe layer → V layer at its transition. This might explain the slight asymmetry of the concentration slope for both at 603 K and 297 K; the intermixing at the V/Fe sequence is less pronounced than that at the Fe/V sequence. However, the slope of *c*_Fe_ at Fe/V interface is considerably steeper in 297 K compared to 603 K (compare Figure 4 and Figure 5). This clearly indicates the impact of the deposition temperature, apart from the sole impact of the *E_F_* difference. In addition, as the films were prepared by sputter deposition, the origin of this asymmetry may be associated with sputter induced implantation and recoil phenomena as discussed below.

In order to investigate the collision details of the sputtering process, simulations were carried out with SRIM 2008 developed by Ziegler [35]. At first, simulations of the Ar ion (880 eV) bombardment into V and Fe were carried out to set the kinetic energy of the sputtered V and Fe ions as *E*_V_ = 33.01 eV and *E*_Fe_ = 24.81 eV, respectively. For each collision calculation, a reasonable displacement energy (Wigner energy) value for metals of 24 eV for target materials was assumed [36]. These parameters were then applied in the simulation.

**Figure 6:**
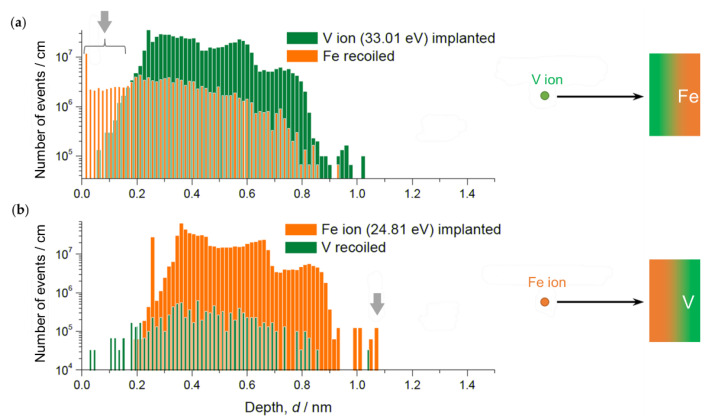
SRIM simulation results of implantation depths and recoil distributions, for V in Fe (**a**) and Fe in V (**b**). Note significant recoil of Fe and deeper penetration depth of Fe than that of V (indicated by grey arrows).

Figure 6 shows simulated ion implantation depth profiles and target atom recoil profiles for Fe ions impinging an V/Fe double layer and for V ions impinging an Fe/V double layer, respectively. According to these results, the following main insights can be extracted.

(1) The maximum implantation depth of Fe is slightly deeper than that of V.
(2) The intensive recoil events of Fe indicate (first 0.1 nm in Figure 6a) floating Fe at the deposition front.
(3) The recoil of V is much less pronounced than that of Fe (recoiled V ion count is one order of magnitude smaller).

(1) explains the slightly wider reaching interface of Fe/V compared to that of V/Fe, regardless of the temperature. (2) and (3) suggest continuous motion of Fe or V atoms towards the deposition front which may increase the intermixing layer thickness stronger than the values simulated here. This process might change also the layer composition if the temperature is high and solubility is ensured to a certain extent.

Moreover, especially in the sequence of V on Fe, Fe atoms propagate always towards the deposition front due to the recoil and substantial alloying with V if the deposition temperature is high enough. The same process is possible also for the deposition of Fe onto V, but very suppressed due to small kinetic energy of Fe ion to recoil V. A continuous propagation of the floating Fe atoms finally stops at the Pd surface, which, then, terminates with a considerable amount of Fe in Pd (see Figure 1 and Figure 2).

**Figure 7:**
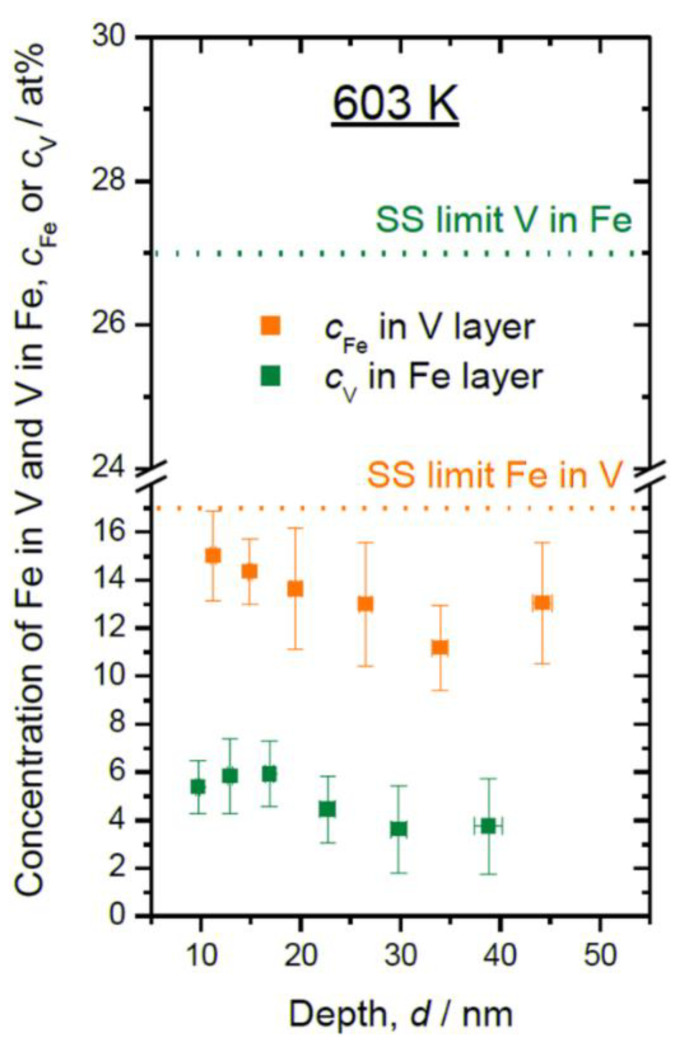
In-layer composition of Fe in V layers (*c*_Fe_) and V in Fe layers (*c*_V_) plotted against the sample depth, as detected at 603 K. Solid solubility limits (SS limit) at 603 K for bulk system [31] are marked with dotted lines.

Consequently, the layer composition of the sample deposited at 603 K is also affected by the sputtering process. In Figure 7, the in-layer compositions are plotted for the film deposited at 603 K, together with the solid solubility limit (SS limit) at the same temperature. Slightly gradual slopes of in-layer Fe concentration (*c*_Fe_) and V concentration (*c*_V_) along the deposition direction suggest the trace of recoiled atoms and high degree of alloying at 603 K.

#### 2.1.3. Combined Effect of Sputtering and Thermal Interdiffusion

From the results obtained, a schema of Fe/V multi-layer deposition can be drawn as follows. The primary knocked-on Fe or V atoms are deposited not only onto the surface, but also in a sample depth. A series of this process, i.e., sputter deposition, creates new intermixed surface. Consequently, the actual intermixing width becomes slightly wider than the values calculated on a single process as shown in Figure 6.

As a conclusion, the interdiffusion process was incorporated with floating phenomenon of atoms at a high deposition temperature and resulted in higher extent of alloying than at room temperature. Hetero-epitaxy of multi-layered films with high crystal quality is established often at certain high deposition temperatures at a given deposition rate and atmosphere. For Fe/V superlattice growth by magnetron sputtering, which has 10 times higher deposition rate than here, hetero-epitaxy is reported, e.g., at 453 K [37]. However, this was not the case for Fe/V prepared in this study. Therefore, the preparation of Fe/V multi-layered film samples for D_2_ loading shown in the following section was not carried out at elevated temperatures.

### 2.2. D in FeV ML—The Impact of D_2_ Loading Pressure

#### 2.2.1. D_2_ 0.05 Pa

D_2_ loading was carried out at various D_2_ pressures at 294 K utilizing a home-made D_2_ loading system suitable for APT analysis (see Materials and Methods).

**Figure 8:**
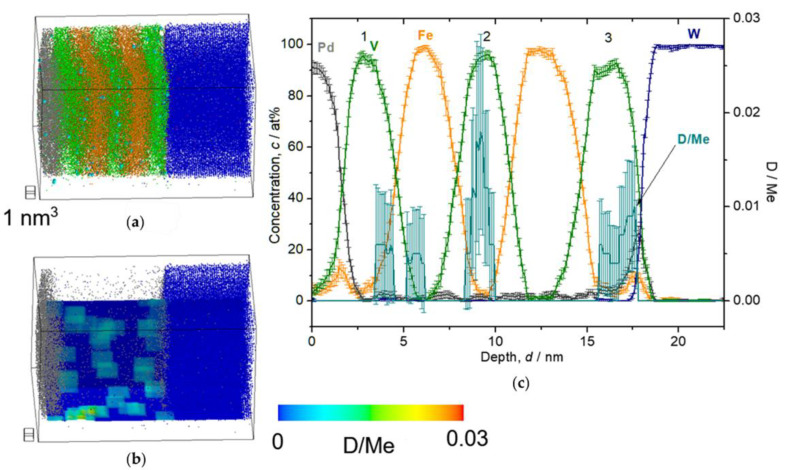
Reconstructed volume of Fe/V multi-layer (14 nm × 14 nm × 27 nm), loaded with D_2_ 0.05 Pa, analyzed at 30 K. (**a**) The whole volume of reconstruction (grey: Pd, green: V, orange: Fe, blue: W, light blue: D). (**b**) Iso-concentration map of D concentration (*c*_D_ = 0.03 D/Me) from the same reconstruction in (**a**). (**c**) Depth concentration profile from 5 nm *ϕ* cylinder volume.

Following the solubility difference factor *k*, D atoms were detected mainly in V layers, but not in Pd or W (*c*_H in Pd_/*c*_H in V_ = 10^−4^, *c*_H in W_/*c*_H in V_ = 10^−24^ [26]). The detected deuterium concentration (*c*_D_) was alternatively verified by an EMF curve of similarly grown Fe/V ML films. It should be noted that the EMF is taken for H, and slight differences for the two isotopes H and D, are neglected, here. At low D_2_ pressure of 0.05 Pa, we expect the sample to be in the α-phase (deuterium solid solution phase) with *c*_D_ = 0.035 D/Me, according to the EMF curve (cf. [24] or Figure 13). The result of the 3D reconstruction, the iso-concentration map and the depth profile of this sample are shown in Figure 8. The dots shown in Figure 8a,b are individual ions. The color map shown in (b) overlaid on the ion map indicate the *c*_D_ as molar ratio of D/Metal (D/Me) within a defined cube of 2 nm side lengths. The average *c*_D_ of 0.013(4) D/Me was found in the 2nd V layer. This value is only 1/3 of the value expected from the EMF-curve. This can be explained by D trapping at defects which is included in the EMF curve [38,39]. The related EMF curve (later shown in Figure 13) deviates non-linearly from the Sieverts’ relationship (a slope of *RT*/*F*), at low concentrations. This leads to higher mean contents of deuterium in the sample. However, if no dislocation exists in the targeted volume of the APT measurement and thus no considerable trapping is expected, the *c*_D_ at 0.05 Pa should almost follow the Sieverts’ law. Then, the expected concentration would be approximately *c*_D_ = 0.02 D/Me, at 0.05 Pa. This value roughly meets the APT value.

#### 2.2.2. D_2_ 0.5–1000 Pa

**Figure 9:**
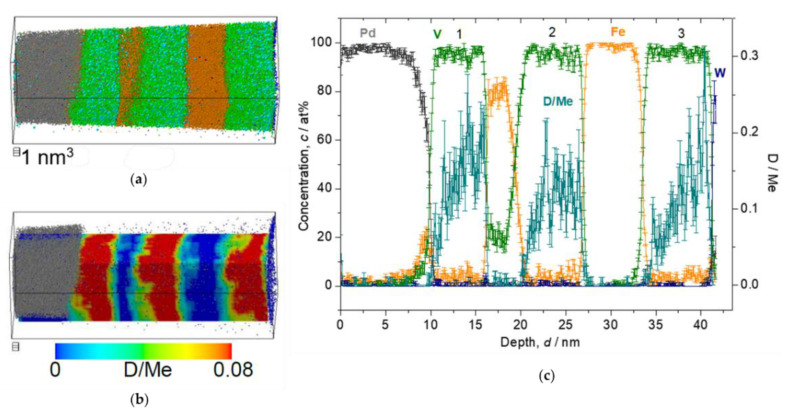
Reconstructed volume of Fe/V multi-layer (15 nm × 15 nm × 42 nm), loaded with D_2_ 0.5 Pa, analyzed at 30 K. (**a**) The whole volume of reconstruction (grey: Pd, green: V, orange: Fe, blue: W, light blue: D). (**b**) Iso-concentration map of D concentration (*c*_D_ = 0.08 D/Me) from the same reconstruction in (**a**). (**c**) Depth concentration profile from 5 nm *ϕ* cylinder volume.

**Figure 10:**
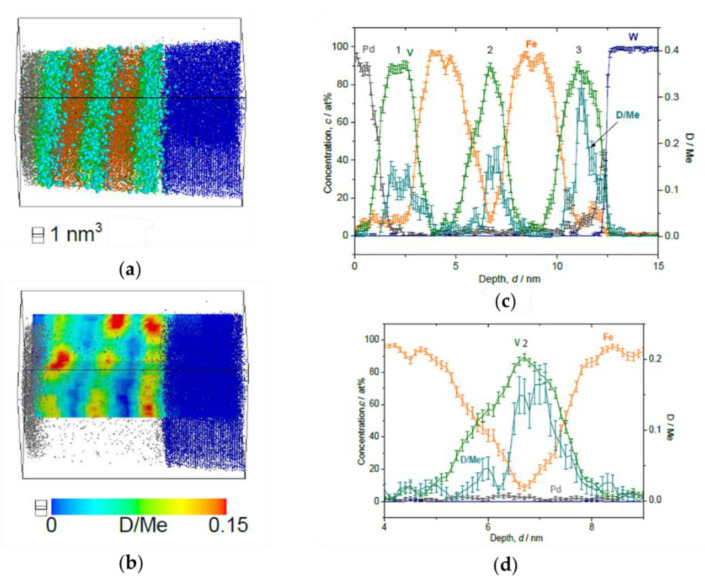
Reconstructed volume of Fe/V multi-layer (12 nm × 12 nm × 20 nm), loaded with D_2_ 2 Pa, analyzed at 30 K. (**a**) The whole volume of reconstruction (grey: Pd, green: V, orange: Fe, blue: W, light blue: D). (**b**) Iso-concentration map of D concentration (*c*_D_ = 0.15 D/Me) from the same reconstruction in (**a**). (**c**) Depth concentration profile from 5 nm *ϕ* cylinder volume. Notably high *c*_D_ in the 3rd V layer is observed. (**d**) Magnified plot of the 2nd V layer. The *c*_D_ shows an asymmetric profile.

**Figure 11:**
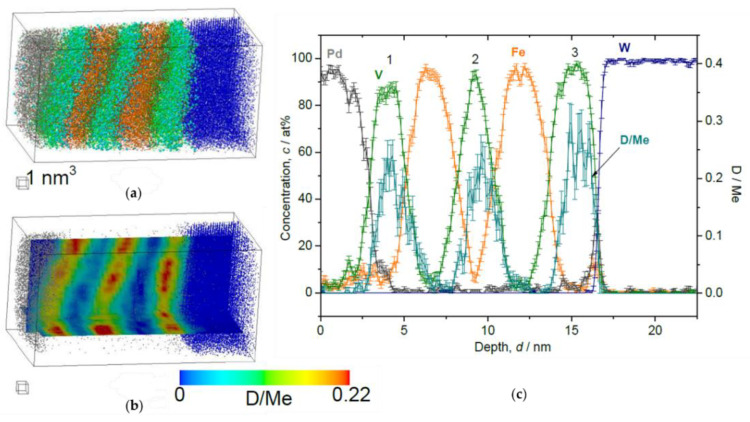
Reconstructed volume of Fe/V multi-layer (12 nm × 12 nm × 23 nm), loaded with D_2_ 10 Pa, analyzed at 30 K. (**a**) The whole volume of reconstruction (grey: Pd, green: V, orange: Fe, blue: W, light blue: D). (**b**) Iso-concentration map of D concentration (*c*_D_ = 0.22 D/Me) from the same reconstruction in (**a**). (**c**) Depth concentration profile from 5 nm *ϕ* cylinder volume. The *c*_D_ in the 3rd V is higher than in the other 2 V layers.

**Figure 12:**
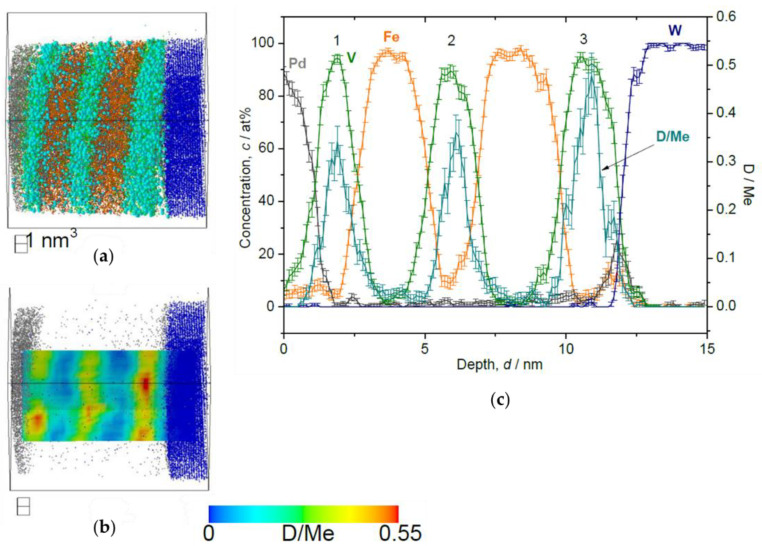
Reconstructed volume of Fe/V multi-layer (12 nm × 12 nm × 16 nm), loaded with D_2_ 1000 Pa, analyzed at 30 K. (**a**) The whole volume of reconstruction (grey: Pd, green: V, orange: Fe, blue: W, light blue: D). (**b**) Iso-concentration map of D concentration (*c*_D_ = 0.55 D/Me) from the same reconstruction in (**a**). (**c**) Depth concentration profile from 5 nm *ϕ* cylinder volume.

When the loading pressure increases from 0.5 to 1000 Pa, *c*_D_ in the V layer increases, as shown in the Figure 9, Figure 10, Figure 11 and Figure 12. In Figure 9, the film structure itself was not well deposited and the 2nd V layer is forming sloped interface with respect to the 1st Fe layer. Nevertheless, it clearly reveals laterally homogeneous D distribution in the V layers, while showing sloped D profile towards the W substrate. The mean *c*_D_ in the 2nd V layer is 0.12(5) D/Me.

At the D_2_ pressure of 2 Pa (Figure 10), the sample is supposed to be in the β-phase (hydride phase: V_2_H) region for bulk V and the observed *c*_D_ = 0.13(5) D/Me reaches the expected average concentration *c*_D_ = 0.15 D/Me of the EMF measurement, within the error bar. Here, one should note that distinctively high *c*_D_ is found in the 3^rd^ V layer just close to W, which is almost twice as large as *c*_D_ in the other two V layers. Such enrichment of D was observed also in case of V/Fe single layered film [12]. Moreover, the *c*_D_ profile in the 2nd V layer is asymmetric, and the concentration maximum of *c*_D_ does not agree with the concentration minimum of *c*_Fe_. As a general trend, the peak position of *c*_D_ is slightly shifted towards the W substrate.

This trend is commonly observed in the results of loading both at 10 Pa (Figure 11) and 1000 Pa (Figure 12). Alloying of V with Fe occurring at the Fe/V interface reduces D-solubility. In addition, the stacking order of Fe/V seems to have a remarkable impact on the D distribution concerning the high *c*_D_ region. At 1000 Pa, the special *c*_D_ distribution is even more pronounced than in the results of the 10 Pa sample, indicating that these curious observations of (i) depth shift of *c*_D_ and (ii) high *c*_D_ in the 3rd V layer might be universally related with the deuteride (hydride) formation behavior, since this is observed at high D_2_ pressures.

#### 2.2.3. Comparison with the EMF Curve (Pressure-Composition Isotherm)

**Figure 13:**
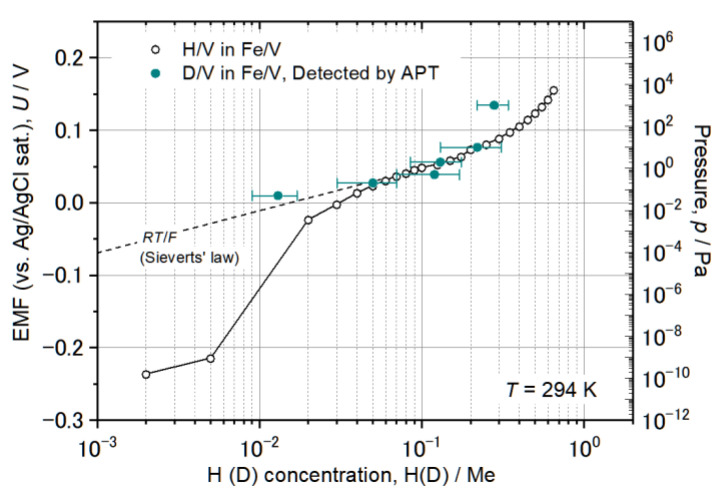
EMF curve of Pd 20 nm/[(Fe 5 nm/V 5 nm) × 8] deposited on Al_2_O_3_ (0001) substrate at room temperature and individual *c*_D_ determined by APT on the [Fe 2–5 nm/V 2–5 nm/Fe 2–5 nm] stack loaded at different D_2_ pressure. The black dotted line *RT*/*F* shows the ideal solubility known as Sieverts’ law.

We now summarize the results of those deuterium concentrations *c*_D_ at different D_2_ pressures obtained by APT, comparing the corresponding EMF curves. Both results are summarized in Table 1 and plotted in Figure 13. All APT data points were recorded at the analysis temperature of 30 K and the mean D concentration *c*_D_ in the 2nd V layer from individual results are listed.

Altogether, the agreement is quite well within the error bar. In the results at 0.05 Pa D_2_, however, the detected D_2_ concentration was *c*_D_ = 0.013 D/Me, which is only 1/3 of the expected value. The expected concentration *c*_D_ is 0.035 D/Me if significant H-trapping effect is taken into account. If no dislocation exists for instance in this small analyzed-volume and thus no considerable D-trapping effect at dislocations is expected, the *c*_D_ at 0.05 Pa should almost follow the linear line indicated as *RT*/*F* in Figure 13. This would give *c*_D_ = 0.02 D/Me. Plotting the observed concentration of *c*_D_ = 0.013(4) D/Me in Figure 4 agrees with this consideration if the slight difference is ignored.

Another large gap between the data points was found in case of 1000 Pa. The deviation of the APT data from the corresponding EMF curve is rather large. *c*_D_ was detected to be only 0.28 D/Me by APT which is nearly half of the concentration expected from the EMF curve. The isotope effect alone cannot explain such a huge difference between *c*_D_ and c_H_ as the phase boundaries of the both are nearly the same in the considered concentration range here [40,41]. A depletion effect [42] cannot explain such a huge gap, especially as this effect is considered to be less pronounced at high hydrogen concentrations [43].

Since a loss of D atoms can be disregarded at this analysis temperature, other factors such as the difference of the V layer thickness from that of the sample taken for the EMF or different lattice strain state and the resulting stress contribution may be ascribed to the narrowed miscibility gap. Due to increased brittleness at low analysis temperatures, the thickness of V layers had to be reduced from 5 nm to, e.g., 2 nm for successful APT analysis. Such a reduction in the thickness often reduces the terminal hydrogen concentration [44]. Thus, the comparison was made between films with layer thicknesses of approximately 2 and 5 nm. The observed gap for the high D concentration regime suggests that even a small difference in the layer thickness might remarkably influence the resultant terminal solubility, especially for high D concentrations. At low concentrations, other aspects might be involved; if defects as D-trapping centers are not included in the analysis volume by chance, the resultant D concentration might be less than that of the defect-containing counterparts, where trapping effects are significant.

Conclusively, the APT analysis of deuterium in Fe/V can be carried out with enough reliability in a wide concentration range of *c*_D_ = 0.01 − 0.2 D/Me at 30 K.

#### 2.2.4. Asymmetric D Profiles at Fe/V and V/Fe Interfaces

The asymmetric D profiles have been already presented in Figure 10, Figure 11 and Figure 12. By taking the *c*_D_ at the 2nd Fe/V interface and plotting it against the Fe concentration from the sequence of V/Fe and Fe/V for comparison, the difference of the D profiles at the two interfaces becomes clearly visible.

**Figure 14:**
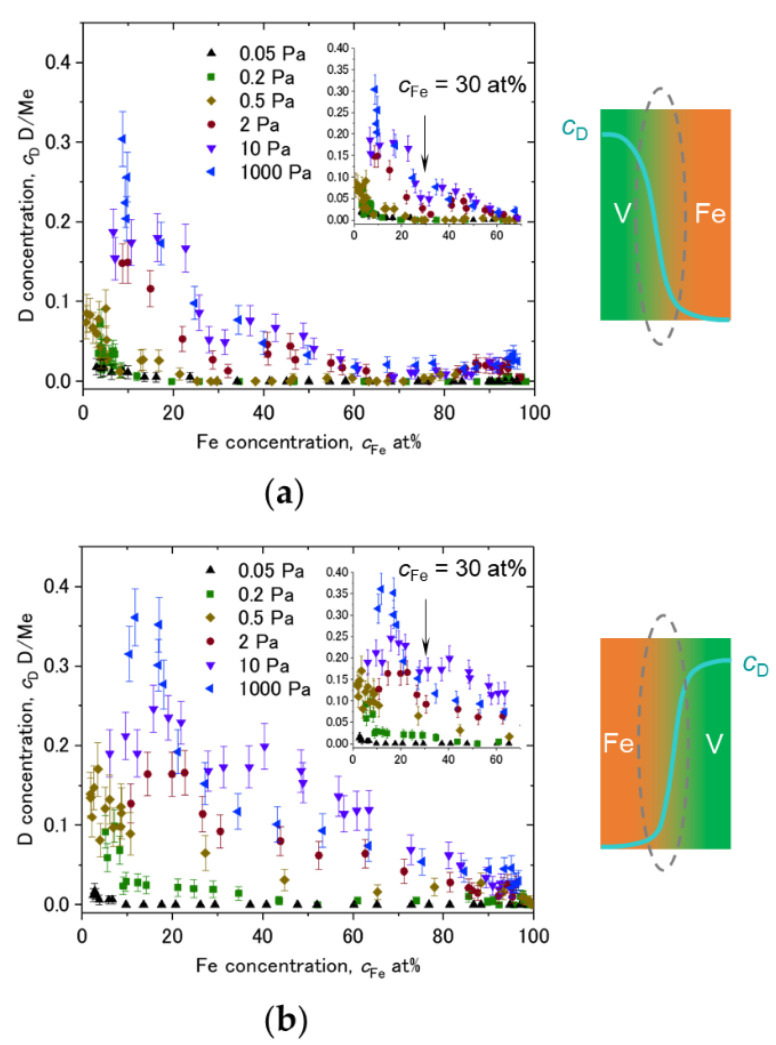

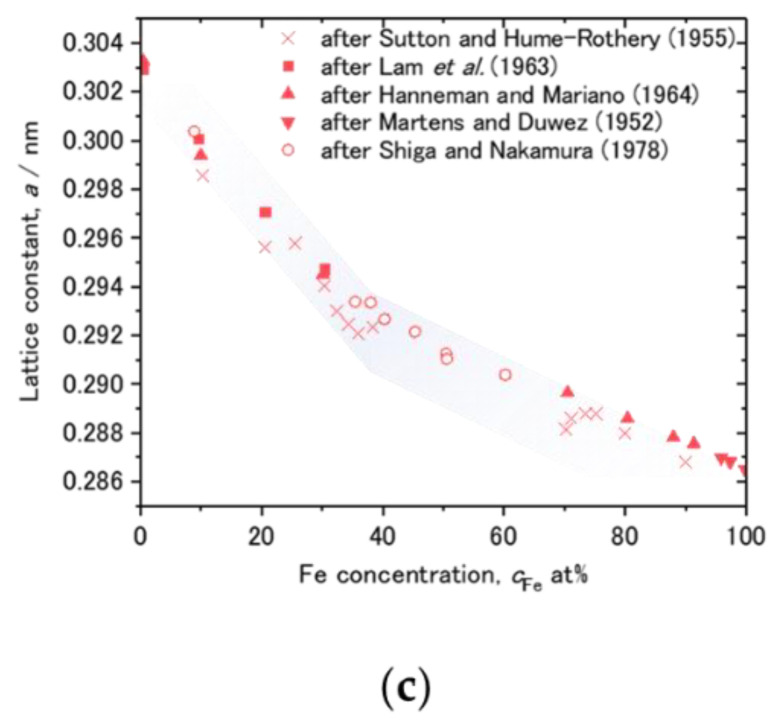
Dependence of *c*_D_ on c_Fe_ at various D_2_ loading pressures, plotted for (**a**) V/Fe interface and (**b**) Fe/V interface at the 2nd V layer of Figure 8, Figure 9, Figure 10, Figure 11 and Figure 12. Schematic drawings of *c*_D_ change with *c*_Fe_ at the intermixed regions are depicted next to the profiles. *c*_Fe_ = 30 at% is marked by black arrows in the insets. (**c**) Change of lattice constant of FeV alloy against Fe concentration, reported by Shiga and Nakamura [46]. Individual data points were taken from Sutton and Hume-Rothery [47], Lam et al. [48], Hanneman and Mariano [49], Martens and Duwez [50], and Shiga and Nakamura [46]. Note the Vegard’s law slope changes at around *c*_Fe_ = 30~35 at%.

Plots of *c*_D_ against *c*_Fe_ taken from *c*_D_ at the Fe/V and V/Fe interfaces are shown in Figure 14a,b, respectively. The corresponding interface is schematically illustrated as well. The *c*_Fe_ range around 15 at% and 65 at% is magnified in the inset. In both of (a) and (b), it is commonly observed that the *c*_D_ decreases as *c*_Fe_ increases. This could be due to an impact of alloying with Fe. Figure 14c presents the lattice parameter of the FeV alloy as function of the Fe-concentration *c*_Fe_. It decreases with increasing *c*_Fe_. Such a change in the lattice parameter is considered to change the available volume for the H atom. Lebon et al. [45] demonstrated by Density Functional Theory (DFT) calculation that alloying of V with Fe, Mn and Cr decreases the volume for H. The result is consistent with the experiments, and also with our experimentally observed general reduction in the D-solubility with increasing the Fe content. As highlighted by black arrows in the insets of Figure 14a,b, there seems to be a discontinuity in the change in *c*_D_ against *c*_Fe_ at around *c*_Fe_ = 30~35 at%. Interestingly, this Fe concentration corresponds to the region where σ-phase is formed in Fe-V phase diagram [31]. Above this concentration range, the slope changes in Figure 14c. This discontinuity detected in the APT profiles visible in the related volumes supports the strong impact of the available volume on the local hydrogen isotope content.

Despite of the same *c*_Fe_, the *c*_D_ dependence on *c*_Fe_ in Figure 14a,b are not identical especially at high D_2_ pressures. The local mean D concentration is higher at the V/Fe interface than at the Fe/V interface. The alloying effect discussed above cannot explain this unidentical profiles in (a) and (b). Additionally, this cannot be related to an increased number of defects such as misfit dislocations, since this is expected to be effective at the Fe/V interface, but not at the V/Fe interface (lattice constant: *a*_Fe_ = 0.286 nm < *a*_V_ = 0.303 nm). Considering the sign of expected misfit edge dislocation, this should result in opposite *c*_D_ profile to the observation. We suggest that different lattice strain is responsible for this difference. However, electronic and stress effects should also be considered. For example, the SRIM simulation results (Figure 6) suggest that the implantation impact of Fe into V and V into Fe is not identical. This might cause a difference in the initial stress state between the Fe/V and V/Fe interfaces. Hydrogen isotopes prefer the less compressive region. It is inferred that the in-plane stress at V/Fe interfaces could be more compressive than at Fe/V interfaces due to the difference in the lattice parameters between Fe and V. This also suggests an expanded V lattice in the out-of-plane direction near the V/Fe interface. Such anisotropic lattice expansion in vicinity of the V/Fe interface, even when alloyed, might change the site occupation of D here, giving rise to different D concentrations even at the same *c*_Fe_ at the alloyed Fe/V interfaces.

## 3. Materials and Methods

### 3.1. Sample Preparation

Fe/V multi-layered films were deposited on needle-shaped substrates suitable for the APT analysis. In this study, we employed W wires with 0.1 mm diameter as substrates since the lattice mismatch between W and V is approximately 4.6%, which is in the same order of magnitude as between Fe and V with 5.3%. Still, this large lattice misfit might induce misfit dislocations implemented at the film/substrate interface.

First, the W tip substrates were sharpened by electropolishing in a 2N NaOH water solution at 3.5 V a.c., by using Pt as counter electrode. Then, the sharpened W tips were finally developed by Field Ion Microscopy (FIM) imaging using He as an imaging gas, typically at 30 K and with approximately 11 kV to obtain smooth and atomically clean surfaces in an UHV chamber. The developed tips have (110) orientation along the tip axis, reflecting the textured bcc structure of the W wire. Fe/V multi-layers with an individual layer thickness of 2~5 nm were deposited on the top of W tips by ion beam sputter deposition in a separate UHV chamber having a base pressure of 1–2 × 10^−8^ Pa. After surface cleaning of W tip by Ar ion for several to 10 s, sputtering was carried out under Ar atmosphere at the pressure of 1 × 10^−2^ Pa, with deposition rates of 0.63 nm/min for V, and 0.75 nm/min for Fe, respectively. The substrate temperature during deposition was kept at 297 K or 603 K. Finally, the films prepared were terminated by an approximately 20 nm thick Pd layer, which facilitates D_2_ dissociation at the surface and protects the film from oxidation.

### 3.2. Deuterium Loading at Different Pressures

**Figure 15:**
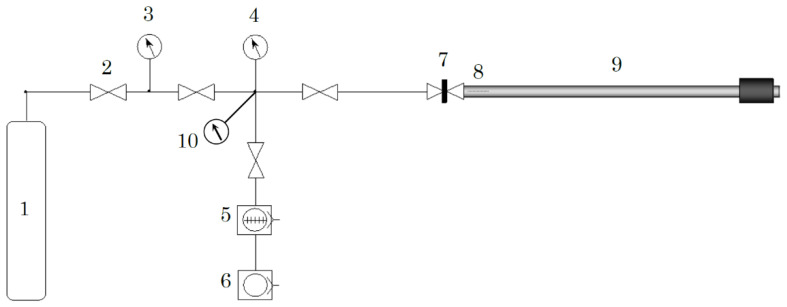
Schematic illustration of the home-made deuterium gas loading set up. 1: D_2_ gas bottle (99.98%), 2: variable leak valve, 3: capacitance manometer 10^5^ Pa max., 4: capacitance manometer 100 Pa max., 5: turbo molecular pump, 6: oil rotary pump, 7: gate valve, 8: sample mounted on transfer rod, 9: magnetic transfer rod with sample, and 10: vacuum gauge.

Deuterium loading was carried out by using a specially constructed gas loading set up, as shown in Figure 15. This system is equipped with a magnetic sample transfer rod, coupled with a gate valve so that the D_2_-loaded sample can be transferred from the loading chamber to APT chamber without breaking D_2_ atmosphere.

The actual D_2_ loading procedure typically starts as follows. First, the whole system was evacuated until the pressure reaches better than 1 × 10^−5^ Pa. Thereafter, bake-out of the transfer rod was carried out at 383 K for 12 h with utilizing ribbon heater elements. Subsequently, the pressure typically showed 10^−6^ Pa at room temperature. Thereafter, deuterium gas (purity: 99.98%) was leaked into the system. The pressure of the D_2_ gas can be measured by capacitance manometer (company: Baratron, MKS) and is kept at a desired pressure just by adjusting the leak valve’s opening. As soon as the D_2_ gas was introduced at a desired pressure, the gate valve for the transfer rod was closed and the sample was loaded with deuterium for 24~48 h.

After loading was completed, the transfer rod was detached from the loading set up together with the gate valve and then connected to a pre-evacuation chamber on the APT. Before introducing the sample into the main chamber, the pre-evacuation chamber was evacuated down to a pressure lower than 6 × 10^−6^ Pa. Then, the gate valve was opened to evacuate also the remaining D_2_ gas from the transfer rod. After breaking D_2_ atmosphere the introduction of sample into the main chamber was completed within 10 min. Upon mounting the sample on cooling stage, the sample was rapidly cooled down to 130 K and, thereafter, to the desired temperature for analysis.

### 3.3. APT Analysis

APT analysis was carried out with a system of tomographic atom probe detector type [4] at 30 K with a voltage pulse fraction of 20% and a voltage pulse frequency of 2 kHz. In particular for analyzing D, low analysis temperatures are mandatory to suppress the D-diffusion [24]. The standing voltage of the sample changed typically from 3.5 to 15 kV to complete an analysis.

After data acquisition, reconstruction of the collected ions to three-dimensional volumes was carried out. Upon reconstruction, the requirement of consideration on the sample geometry is especially strict for multi-layered sample. Jeske and Schmitz [51] have developed a reconstruction algorithm to solve this geometry problem by introducing the factors on the specimen’s initial radius and shaft angle. In this study, their algorithm was employed, and successful reconstruction was achieved.

### 3.4. Verification of c_D_ via EMF Measurement

The average deuterium concentration *c*_D_ detected in the Fe/V multi-layers (on the W tip) and analyzed by APT was compared with the average hydrogen concentration *c*_H_ in Fe/V (110) multi-layered film grown on an Al_2_O_3_ (0001) substrate. Here, *c*_H_ was measured by electrochemical H loading [38]. The related electromotive force (EMF) was detected, giving an EMF curve against *c*_H_. This curve relates to a pressure-composition isotherm (p-c-T curve) via the Nernst equation. Slight differences induced by the different loading techniques are thereby neglected. This experimental technique is advantageous since a well-defined concentration of hydrogen can be loaded stepwise [38] and the p-c-T property around room temperature can be correctly measured even in nano-sized samples. On top of this, gas phase loading is generally difficult for materials such as V, unless an activation process is properly carried out. The positions of the α and α + β phase boundaries are essentially the same in the bulk V–H and V–D systems at 300 K, at *c*_H_, α = 0.03 H(D)/V and *c*_H_, α + β = 0.47 H(D)/V. Similar phase boundary values were found both for bulk [40] and for a thin film with a thickness of 500 nm [41]. This enables direct comparison of *c*_H_ and *c*_D_ as conducted in this study. Electrochemical loading was carried out by using a mixed solution of phosphoric acid 85% and glycerin 85% (1:2 in volume) as an electrolyte, a Ag/AgCl(sat.) and a Pt wire as the reference and the counter electrode, respectively. The *c*_H_ was calculated by using Faraday’s law.

## 4. Summary

In this paper, we investigated concentration profiles of Fe/V interfaces in Fe/V multi-layered films and the D distributions therein by Atom Probe Tomography (APT) analysis. A combined effect was suggested including local alloying at the Fe/V interfaces at high deposition temperatures and an impact of particle recoil during the deposition. The resulting Fe/V interfaces appear asymmetric along the film growth direction; a steeper interface was observed at the Fe/V side compared to the V/Fe side even at a low deposition temperature. This was interpreted by a pronounced implantation of Fe atoms in a deposition sequence of Fe on the V layer.

The mean D concentration in Fe/V ML was determined in a wide D concentration range and compared with a representative electromotive force (EMF) curve. The agreement was satisfactory as long as the impacts of D-trapping at defects and thickness differences are taken into account. With regard to the local alloying at Fe/V interfaces, the dependence of the D concentration on the Fe concentration demonstrated a correlation, reflecting the change in lattice constant, i.e., the change in available volume for D. However, the D distribution was found to follow the asymmetric Fe/V interface compositions especially at high D_2_ pressures. This was considered to originate from different stress states at Fe/V interfaces depending on the stacking sequence because of different implantation impacts. To conclude, in Fe/V multi-layers, the local D content is influenced both by local chemistry and strain, which sensitively reflects the preparation history and the geometry of the samples, as demonstrated by this APT study.

## Figures and Tables

**Table 1:** Comparison of mean D concentration *c*_D_ [D/Me] obtained by the EMF curve and APT.

D_2_ Pressure [Pa]	Mean D Concentration, *c*_D_ [D/Me]	
	Expected by EMF ^1^	Detected by APT ^2^
0.05	0.035	0.013(4)
0.2	0.053	0.05(2) ^3^
0.5	0.08	0.12(5)
2	0.15	0.13(5)
10	0.22	0.22(9)
1000	0.55	0.28(6)

^1^*c*_H_ determined by the EMF curve of Fe/V ML ≈ *c*_D_ (Isotope effect is negligible in the *c*_D_ range here.). ^2^ Mean *c*_D_ in the 2nd V layer (Fe/V/Fe). ^3^ Adapted from our previous report ([24]).

## Data Availability

Not applicable.

## References

[1] Cerezo A., Godfrey T.J., Smith G.D.W. (1998). Application of a Position-sensitive Detector to Atom Probe Microanalysis. Rev. Sci. Instrum..

[2] Blavette D., Cadel E., Fraczkiewicz A., Menand A. (1999). Three-Dimensional Atomic-Scale Imaging of Impurity Segregation to Line Defects. Science.

[3] Miller M.K. (2000). Atom Probe Tomography.

[4] Al-Kassab T., Wollenberger H., Schmitz G., Kirchheim R. (2003). Tomography by Atom Probe Field Ion Microscopy. High-Resolut. Imaging Spectrom. Mater..

[5] Kelly T.F., Miller M.K. (2007). Atom Probe Tomography. Rev. Sci. Instrum..

[6] Gault B., Moody M.P., Cairney J.M., Ringer S.P. (2012). Atom Probe Microscopy.

[7] Larson D.J., Prosa T.J., Ulfig R.M., Geiser B.P., Kelly T.F. (2013). Local Electrode Atom Probe Tomography.

[8] Miller M.K., Forbes R.G. (2014). Atom-Probe Tomography: The Local Electrode Atom Probe.

[9] Sundell G., Thuvander M., Yatim A.K., Nordin H., Andrén H.O. (2015). Direct Observation of Hydrogen and Deuterium in Oxide Grain Boundaries in Corroded Zirconium Alloys. Corros. Sci..

[10] Meier M.S., Jones M.E., Felfer P.J., Moody M.P., Haley D. (2021). Extending Estimating Hydrogen Content in Atom Probe Tomography Experiments Where H2Molecule Formation Occurs. Microsc. Microanal..

[11] Jones M.E., London A.J., Breen A.J., Styman P.D., Sikotra S., Moody M.P., Haley D. (2022). Improving the Quantification of Deuterium in Zirconium Alloy Atom Probe Tomography Data Using Existing Analysis Methods. Microsc. Microanal..

[12] Breen A.J., Stephenson L.T., Sun B., Li Y., Kasian O., Raabe D., Herbig M., Gault B. (2020). Solute Hydrogen and Deuterium Observed at the near Atomic Scale in High-Strength Steel. Acta Mater..

[13] Takahashi J., Kawakami K., Kobayashi Y. (2018). Origin of Hydrogen Trapping Site in Vanadium Carbide Precipitation Strengthening Steel. Acta Mater..

[14] Takamizawa H., Hoshi K., Shimizu Y., Yano F., Inoue K., Nagata S., Shikama T., Nagai Y. (2013). Three-Dimensional Characterization of Deuterium Implanted in Silicon Using Atom Probe Tomography. Appl. Phys. Express.

[15] Shimizu Y., Han B., Ebisawa N., Ichihashi Y., Hashiguchi T., Katayama H., Matsumoto M., Terakawa A., Inoue K., Nagai Y. (2020). 3D Impurity Profiles of Doped/Intrinsic Amorphous-Silicon Layers Composing Textured Silicon Heterojunction Solar Cells Detected by Atom Probe Tomography. Appl. Phys. Express.

[16] Shimizu Y., Sai H., Matsui T., Taki K., Hashiguchi T., Katayama H., Matsumoto M., Terakawa A., Inoue K., Nagai Y. (2020). Crystallite Distribution Analysis Based on Hydrogen Content in Thin-Film Nanocrystalline Silicon Solar Cells by Atom Probe Tomography. Appl. Phys. Express.

[17] Yatagai K., Shishido Y., Gemma R., Boll T., Uchida H.H., Oguri K. (2020). Mechanochemical CO_2_ Methanation over LaNi-Based Alloys. Int. J. Hydrogen Energy.

[18] Gemma R., Al-Kassab T., Kirchheim R., Pundt A. (2011). Analysis of Deuterium in V-Fe5at.% Film by Atom Probe Tomography (APT). J. Alloys Compd..

[19] Takahashi J., Kawakami K., Kobayashi Y., Tarui T. (2010). The First Direct Observation of Hydrogen Trapping Sites in TiC Precipitation-Hardening Steel through Atom Probe Tomography. Scr. Mater..

[20] Chen Y.S., Haley D., Gerstl S.S.A., London A.J., Sweeney F., Wepf R.A., Rainforth W.M., Bagot P.A.J., Moody M.P. (2017). Direct Observation of Individual Hydrogen Atoms at Trapping Sites in a Ferritic Steel. Science.

[21] Chen Y.S., Bagot P.A.J., Moody M.P., Haley D. (2019). Observing Hydrogen in Steel Using Cryogenic Atom Probe Tomography: A Simplified Approach. Int. J. Hydrogen Energy.

[22] Stephenson L.T., Szczepaniak A., Mouton I., Rusitzka K.A.K., Breen A.J., Tezins U., Sturm A., Vogel D., Chang Y., Kontis P. (2018). The Laplace Project: An Integrated Suite for Preparing and Transferring Atom Probe Samples under Cryogenic and UHV Conditions. PLoS ONE.

[23] Felfer P., Ott B., Monajem M., Dalbauer V., Heller M., Josten J., Macaulay C. (2022). An Atom Probe with Ultra-Low Hydrogen Background. Microsc. Microanal..

[24] Gemma R., Al-Kassab T., Kirchheim R., Pundt A. (2009). APT Analyses of Deuterium-Loaded Fe/V Multi-Layered Films. Ultramicroscopy.

[25] Gemma R. (2011). Hydrogen in V-Fe Thin Films and Fe/V-Fe Multi-Layered Thin Films.

[26] Fromm E., Gebhardt E. (1976). Gase Und Kohlenstoff in Metallen.

[27] Yagi E., Kobayashi T., Nakamura S., Kano F., Watanabe K., Fukai Y., Koike S. (1986). Direct Evidence of Stress-Induced Site Change of H in V Observed by the Channeling Method. Phys. Rev. B.

[28] Pálsson G.K., Wälde M., Amft M., Wu Y., Ahlberg M., Wolff M., Pundt A., Hjörvarsson B. (2012). Hydrogen Site Occupancy and Strength of Forces in Nanosized Metal Hydrides. Phys. Rev. B Condens. Matter Mater. Phys..

[29] Johansson R., Ahuja R., Eriksson O., Hjörvarsson B., Scheicher R.H. (2015). Effect of Uniaxial Strain on the Site Occupancy of Hydrogen in Vanadium from Density-Functional Calculations. Sci. Rep..

[30] Birch J., Yamamoto Y., Hultman L., Radnoczi G., Sundgren J.E., Wallenberg L.R. (1990). Growth and Structural Characterization of Single-Crystal (001) Oriented Mo/V Superlattices. Vacuum.

[31] Ivanchenko V. (2008). Fe-V (Iron-Vanadium) Phase Diagram Crystal Structure. Landolt Börnstein.

[32] Marquis E.A., Vurpillot F. (2008). Chromatic Aberrations in the Field Evaporation Behavior of Small Precipitates. Microsc. Microanal..

[33] Torres K.L., Geiser B., Moody M.P., Ringer S.P., Thompson G.B. (2011). Field Evaporation Behavior in [0 0 1] FePt Thin Films. Ultramicroscopy.

[34] Brons J.G., Herzing A.A., Henry K.T., Anderson I.M., Thompson G.B. (2014). Comparison of Atom Probe Compositional Fidelity across Thin Film Interfaces. Thin Solid Film..

[35] Ziegler J.F. (1985). The Stopping and Range of Ions in Solids/J.F. Ziegler, J.P. Biersack, U. Littmark.

[36] Haasen P., Mordike B.L. (1996). Physical Metallurgy.

[37] Isberg P., Granberg P., Svedberg E.B., Hjörvarsson B., Wäppling R., Nordblad P. (1998). Structure and Magnetic Properties of Fe/V (110) Superlattices. Phys. Rev. B.

[38] Kirchheim R. (1988). Hydrogen Solubility and Diffusivity in Defective and Amorphous Metals. Prog. Mater. Sci..

[39] Pundt A., Kirchheim R. (2006). Hydrogen in Metals: Microstructural Aspects. Annu. Rev. Mater. Res..

[40] Schober T., Wenzl H. (1978). Systems NbH(D), TaH(D), VH(D): Structures, Phase Diagrams, Morphologies, Methods of Preparation. Hydrog. Met 2.

[41] Papathanassopoulos K., Wenzl H. (1982). Pressure-Composition Isotherms of Hydrogen and Deuterium in Vanadium Films Measured with a Vibrating Quartz Microbalance. J. Phys. F Met. Phys..

[42] Gemma R., Al-Kassab T., Kirchheim R., Pundt A. (2012). Visualization of Deuterium Dead Layer by Atom Probe Tomography. Scr. Mater..

[43] Olafsson S., Hjörvarsson B., Stillesjö F., Karlsson E., Birch J., Sundgren J.E. (1995). Charge Transfer at Interfaces in MoxV1−x/V Superlattices. Phys. Rev. B.

[44] Wagner S., Pundt A. (2016). Quasi-Thermodynamic Model on Hydride Formation in Palladium–Hydrogen Thin Films: Impact of Elastic and Microstructural Constraints. Int. J. Hydrogen Energy.

[45] Lebon A., Vega A., Mokrani A. (2010). Ab Initio Study of Hydrogen Insertion in Ultrathin Transition Metal Doped v Films: Structural and Electronic Properties. Phys. Rev. B Condens. Matter Mater. Phys..

[46] Shiga M., Nakamura Y. (1978). Effect of Local Environment on Formation of Local Moments in BCC Fe-V Alloys: Mossbauer Study. J. Phys. F Met. Phys..

[47] Sutton A.L., Hume-Rothery W. (2010). CXLI. The Lattice Spacings of Solid Solutions of Titanium, Vanadium, Chromium, Manganese, Cobalt and Nickel in α-Iron. Lond. Edinb. Dublin Philos. Mag. J. Sci..

[48] Lam D.J., Van Ostenburg D.O., Nevitt M.V., Trapp H.D., Pracht D.W. (1963). Magnetic Susceptibilities and Nuclear Magnetic Resonance Measurements in V-Fe Alloys. Phys. Rev..

[49] Hanneman R.E., Mariano A.N. (1964). Lattice-Parameter and Volumetric Data of Iron-Vanadium System. Trans. Met. Soc. AIME.

[50] Martens H., Duwez P. (1952). Phase Relationships in the Iron-Chromium-Vanadium System. Trans. ASM.

[51] Jeske T., Schmitz G. (2002). Influence of the Microstructure on the Interreaction of Al/Ni Investigated by Tomographic Atom Probe. Mater. Sci. Eng. A.

